# Design of 3D Virtual Reality in the Metaverse for Environmental Conservation Education Based on Cognitive Theory

**DOI:** 10.3390/s22218329

**Published:** 2022-10-30

**Authors:** Shih-Che Lo, Hung-Hsu Tsai

**Affiliations:** 1 Department of Industrial Management, National Taiwan University of Science and Technology, Taipei 106335, Taiwan; 2 Institute of Data Science and Information Computing, National Chung Hsing University, Taichung 402204, Taiwan

**Keywords:** virtual reality, cognitive theory, interactive VR portfolios, metaverse

## Abstract

Background: Climate change causes devastating impacts with extreme weather conditions, such as flooding, polar ice caps melting, sea level rise, and droughts. Environmental conservation education is an important and ongoing project nowadays for all governments in the world. In this paper, a novel 3D virtual reality architecture in the metaverse (VRAM) is proposed to foster water resources education using modern information technology. Methods: A quasi-experimental study was performed to observe a comparison between learning involving VRAM and learning without VRAM. The 3D VRAM multimedia content comes from a picture book for learning environmental conservation concepts, based on the cognitive theory of multimedia learning to enhance human cognition. Learners wear VRAM helmets to run VRAM Android apps by entering the immersive environment for playing and/or interacting with 3D VRAM multimedia content in the metaverse. They shake their head to move the interaction sign to initiate interactive actions, such as replaying, going to consecutive video clips, displaying text annotations, and replying to questions when learning soil-and-water conservation course materials. Interactive portfolios of triggering actions are transferred to the cloud computing database immediately by the app. Results: Experimental results showed that participants who received instruction involving VRAM had significant improvement in their flow experience, learning motivation, learning interaction, self-efficacy, and presence in learning environmental conservation concepts. Conclusions: The novel VRAM is highly suitable for multimedia educational systems. Moreover, learners’ interactive VRAM portfolios can be analyzed by big-data analytics to understand behaviors for using VRAM in the future to improve the quality of environmental conservation education.

## 1. Introduction

It is estimated that around 5 billion people, out of a total population of around 8 billion worldwide, will be suffering water-related impacts by 2025 [1]. The main climate change outcomes related to water resources are increases in water temperature, polar ice caps melting, sea level rise and shifts in precipitation patterns, and a likely increase in the frequency of flooding and shortage of water resources. There are some government-created and private institutes around the world aiming to provide water resources education, such as the USGS Water Science School in the US, the Department of the Interior and Water Resources Education Program of the Watershed Council, Michigan, and Project Wet Canada—a youth water education program of the Canadian Water Resources Association.

Virtual reality (VR) is a simulated experience that through hardware and software integration can be like or completely different from the real world. It allows users to interact inside a simulated environment constructed by computer programs. VR technology can create a special region that mimics that of the real world via providing functions such as stereoscopic vision, hearing, touching, and experiencing [2,3]. VR technology is growing to be intensively popular and widespread as the metaverse ushers in a new era of digital connectivity. It can be effectively used in various subjects, such as education, business, gaming, medicine, new employee training, entertainment, social networking, tour guiding, etc. [4,5,6].

Several articles have proposed improving education effectiveness via using computer simulations with VR technology. Regarding safety training with VR technology, several papers have proposed building virtual 3D environments for safety education [7,8,9]. The authors of [7] proposed an immersive VR approach using a simulator developed by VR technology for producing a serious game. Users wear a head-mounted display (HMD) with a simulator for training passengers about aviation safety. The HMD-based simulation provides players with the goal of surviving a dangerous aircraft emergency. The authors of [8] presented immersive VR simulators that allowed players to experience dangerous working environments to prevent and reduce accidents in these working environments. The authors of [9] proposed a VR simulation of a fire evacuation with multiple possible exits. The simulation allows players to experience the fire environment for the presence of visible fire and smoke. It also allows players to choose the right egress routes.

A VR teleoperation system has been developed for mimicking robot controls [10]. The system shows visual content with a first-person view during training robot controls. VR visualization content helps players to be operators. A 3D immersive simulation with VR technology has been proposed to promote teachers’ comprehension of a toddler’s consciousness [11]. Caregivers who work with toddlers wear the HMD to play the 3D immersive simulation. A mobile application including a simulation in a 3D virtual environment was developed by the authors of [12]. Learners wear the cardboard-based VR headsets with a smartphone and then enter the simulation to improve musical genre learning in primary education. The application allows learners to enter a 3D virtual environment and move their bodies while listening to music.

Although the above articles presented useful 3D immersive simulations with VR technology for players, the design of content players watch/operate in the simulation environment seems to take rare consideration of applying the cognitive theory of multimedia learning (CTML) [13,14,15,16,17]. While making multimedia VR content based on CTML, multimedia content can offer more potential explanations [18].

The Soil and Water Conservation Bureau (SWCB) of Taiwan strongly promotes educational concepts for water and soil conservation for Taiwan’s younger generation. The SWCB has produced several children’s color books in both hard copy and soft copy. The SWCB would like not only to quickly distribute educational books conveying important concepts for environmental protection but also make these materials more fascinating for readers, especially for younger generations. This paper proposes a novel 3D virtual reality architecture in the metaverse (VRAM) that applies VR technology in producing a 3D VR simulation for reading a picture book. The 3D VRMA includes 3D VR object animation and interactions between players and content and can be operated using a mobile application, such as an Android app. Learners first put the smartphone into an affordable VR helmet and then wear the helmet to start watching the 3D VRAM course materials anywhere in the world. The 3D VRAM course materials are realized by 3D VR multimedia content in this paper based on CTML for improving learners’ perception of multimedia presentations. One of the main features of VRAM is to offer interactions between players and content. This allows collection of interaction portfolios of users. In the future, these interaction portfolios can be analyzed by big-data analytics for recognizing users’ behavior in utilizing the VRAM. Table 1 presents a comparison for a color book as course material implemented by a high-computation 3D VR production book and 3D VR color book with APP.

The VRAM exploits explanatory 3D VR content to effectively exhibit relationships among topics of content to construct deeper perception. The most important applications of VRAM are for learners to feel like they are “inside” water source protection areas, such as restricted reservoir areas or the North Pole to see climate change animation as well as dangerous places, such as landslides and rockslides. In the VRAM framework, an Android app was created to play 3D VR multimedia content for learning basic concepts of water and soil conservation. When learners wear the VR helmet to watch the 3D VR video, shaking their heads can move the interaction sign, a double circle, to superimpose it on the interaction icons (icons are implemented by graphic objects or symbols) to carry out interactions. Once VRAM recognizes a trigger event for interactive actions, it performs the corresponding interaction. These interactions include replaying a video, going to the next video clip, going to the previous video clip, displaying text annotations, presenting questions, and replying to questions. A quasi experiment was also conducted in the classroom to observe a comparison between involving and not involving VRAM for learning.

This paper is organized as follows. Section 2 provides the theoretical background and related works. In Section 3, the design of VRAM is proposed to build the elements of the system. Section 4 describes experiments and results. Section 5 is the discussion of the experiments and results. Finally, Section 6 gives the conclusions of the study.

## 2. Theoretical Background and Related Works

Nowadays, performing multimedia presentations via computers is a cutting-edge technology. It has been widely utilized in various applications [19]. Multimedia can be represented by the combination of words and pictures. Words can be written or spoken. Pictures can be any graphical format, such as illustrations, videos, photos, and animation [16]. Multimedia-learning theory suggests that humans learn more deeply with words and pictures than with words or pictures alone [13,14,20]. Additionally, CTML explains deeply more potential information, which is based on three critical assumptions: dual channels (auditory and visual) for processing information, a limited channel capacity, and an active process of filtering, selecting, organizing, and integrating information for learning [15,17,21]. First, people have two individual channels that process auditory/verbal and visual/pictorial information [22]. Second, people process each channel with a limited amount of information at one time [23]. Third, people engage in active learning via selecting relevant incoming information, organizing selected information into coherent mental representations, and integrating mental representations with others’ knowledge [24]. Based on these assumptions, CTML postulates principles for guidelines for devising effective multimedia instructions [14]. Learning in VR environments can obtain deeper understanding of the relationship between multimedia learning content based on CTML and learning effectiveness. Each learner can be an active participant who builds a coherent mental representation and then forms new knowledge. The authors of [17] designed 3D VR multimedia content based on a subset of principles of CTML. Applying principles to the design of 3D VR multimedia content is briefly described as follows.

1. Multimedia Principle: People learn better from words plus pictures than from words alone. Our proposed VRAM implements this principle by developing 3D VR multimedia content (video and animation formats for 3D VR objects) and also overlaying texts or icons on 3D VR objects of video and by augmenting graphs with text for performing interactions (e.g., displaying interaction icons on 3D VR video for guidance in interaction between players and 3D VR multimedia content). VR multimedia content consists of auditory information, annotation with text, and explanatory visuals that convey relationships among VR multimedia content topics so as to enhance deeper cognition.
2. Spatial and Temporal Contiguity Principles: People learn better when corresponding words and pictures are contiguously presented, rather than successively presented far from each other on the page or screen. Our proposed VRAM implements contiguity principles by displaying related 3D VR objects and simultaneously superimposing interaction icons and text annotations on the same screen. This realizes spatial and temporal alignment-related 3D VR content and annotation information.
3. Coherence Principle: People learn better when extraneous words, pictures, and sounds are excluded rather than included. Our proposed VRAM can implement this principle via developing 3D VR multimedia content in a storytelling fashion, thereby excluding extra content not related to the story.
4. Signaling Principle: People learn better when cues that highlight the organization of essential information are added. Our proposed VRAM implements this principle by directly guiding players to interact with a trigger event and carry out corresponding actions, including replaying, going to the next video, going to the previous video, displaying text annotations, presenting questions, and replying to questions.
5. Segmentation Principle: People learn better from a multimedia lesson that is presented in user-paced segments rather than as a continuous unit. Our proposed VRAM implements this principle by breaking 3D VR multimedia content down into small video clips (a video clip for a page of the hard-copy version of the picture book) that can be processed at a player’s preferred rate via operating left or right arrow icons. Additionally, a replay icon on 3D VR content executes the segmentation of short logical start/stop controls.
6. Redundancy Principle: People learn better from graphics and narration than from graphics, narration, and on-screen text. Our proposed VRAM implements this principle by simultaneously presenting 3D VR animation content and spoken narration (human voices) in a storytelling fashion.
7. Modality Principle: People learn better from graphics and narration than from animation and on-screen text. Our proposed VRAM implements this principle by simultaneously presenting auditory information (narrator storytelling) and displaying animation of 3D VR objects.
8. Voice Principle: People learn better when the narration in multimedia lessons is spoken in a friendly human voice rather than a machine voice. Our proposed VRAM implements this principle by presenting spoken narration with human voices.

Research results reflected that presenting VR multimedia content based on CTML results in an increase in learning performance on problem-solving skills [14]. Several articles have implemented educational VR multimedia content based on the principles of CTML [19,25,26,27,28]. The results of these articles showed learning VR multimedia content-based CTML can build more profound comprehension to improve learning effectiveness. Table 2 presents an overview of these studies investigating the effectiveness of learning VR multimedia content-based CTML.

The authors of [25] developed 2D Java applets and Java 3D virtual reality simulations for learning mathematical manipulatives (VRMM). They showed that students who learned addition and subtraction operations using VR were superior to those who learned mathematic manipulatives via 2D Java applets. The authors of [26] presented an implementation of 3D animation-based cognitive theory of multimedia learning (ACTML). Three principles of CTML—temporal contiguity, personalization, and multimedia principles—were followed in designing materials. The authors of [27] compared the instructional effectiveness of learning from media involving immersive virtual reality and a desktop slideshow for teaching scientific knowledge. The results reflected that the VR simulation had better effectiveness than the PowerPoint lesson. The authors of [19] reviewed computer-mediated reality (CMR) technologies, such as virtual reality, augmented reality (AR), and mixed reality, and proposed an enhancing language education with artificial neo-platform (eLEARN). According to the CTML, CMR with AR/VR offers a new space for learners that can be realized through virtual environments based on perceptions of experiences. Therefore, persons’ experiences can be transferred to form information through an immersive virtual environment. The results of [28] showed that participants who took a lesson with immersive VR based on pretraining principles learned more effectively than when they watched a video.

## 3. The Design of VRAM

**Figure 1:**
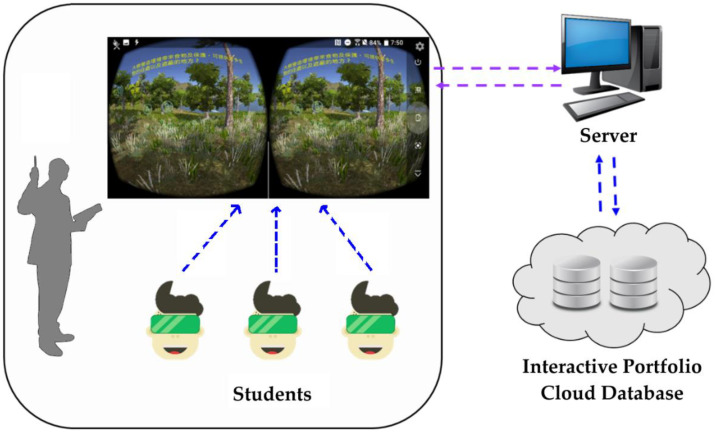
An experimental environment.

Figure 1 shows an experimental environment involving VRAM in learning for water and soil conservation. Each student first puts their smartphone with an Android app into a VR helmet and then wears the VR helmet for playing/watching 3D VR multimedia content. Meanwhile, they can move the interaction sign by superimposing it on interaction icons (graphic symbols). VRAM recognizes the trigger event to perform corresponding interactive actions, such as replaying current a 3D VR video clip, going to the next video clip, going to the previous video clip, showing text annotations, displaying questions, and giving correct answers to the questions. These interaction–action portfolios, watching/playing interactive records, can be immediately sent to the cloud by the app with web services over networks.

### 3.1. Objects of 3D VR

**Figure 2:**
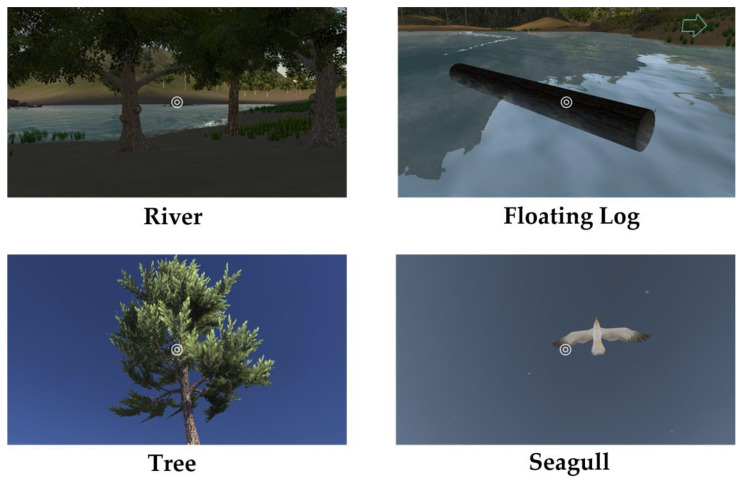
3D VR objects.

**Figure 3:**
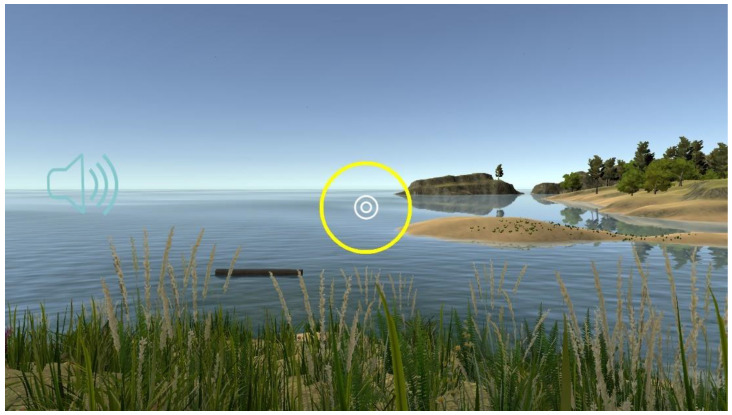
The interaction sign is represented by the white double circle.

3D VR objects in the 3D VR multimedia content are programed by 3dsMax software. These objects are then combined with Unity programs to have animation effects. Figure 2 displays four examples of 3D VR objects: river, floating log, tree, and seagull. Here, the interaction sign is represented by a white double circle, as shown in Figure 3. Its function is like that of using mouse buttons on computers. Players move the interaction sign by slowly shaking their heads while wearing the VR helmet with smartphones.

### 3.2. System Architecture

**Figure 4:**
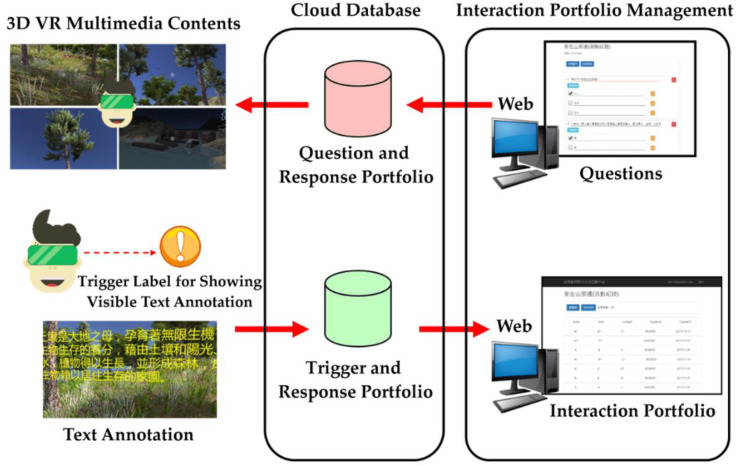
Summary of how to merge quizzing materials into the VRAM system.

Figure 4 exhibits a summary of how to merge quizzing materials into the VRAM system, with four main components: an Android app, a video with 3D VR multimedia content, the cloud database, and interaction portfolio management. The descriptions of the design of the Android app and the video with 3D VR multimedia content are given in the next subsections. The cloud database and interaction portfolio management are stated as follows.

**Figure 5:**
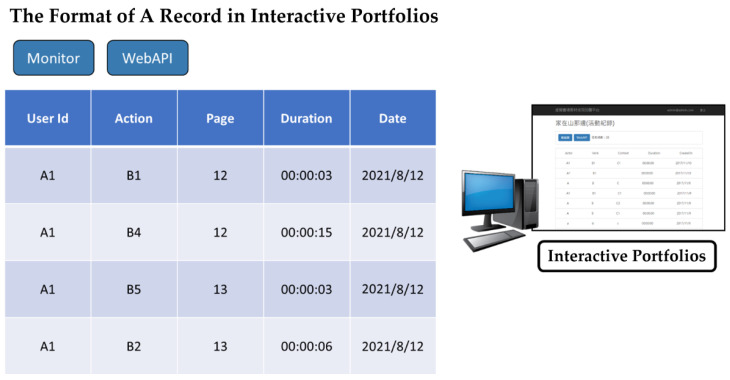
Each record in the interactive portfolio with five fields in xAPI format.

Once players trigger interactive actions, the app delivers corresponding records to the cloud database to construct two kinds of interactive portfolios: the question-and-response portfolio and the trigger-and-response portfolio. The format of each interactive record is created by Experience API (xAPI) [29]. For the case of “going to page,” Figure 5 displays each record using five fields: User Id, Verb, Page, Duration, and Date. The benefit of adopting xAPI for recording interactive data is that it is a lightweight way to store and retrieve records about learners’ experience data and to compatibly share these data across platforms. Subsequently, the interaction portfolio management component provides two operations, maintaining questions linked to their correct answers and displaying the statistical results of players’ responses to questions. A monitor button in Figure 5 is utilized to visually display the records on-screen in real time.

### 3.3. 3D VR Multimedia Content and Interactive Actions

**Figure 6:**
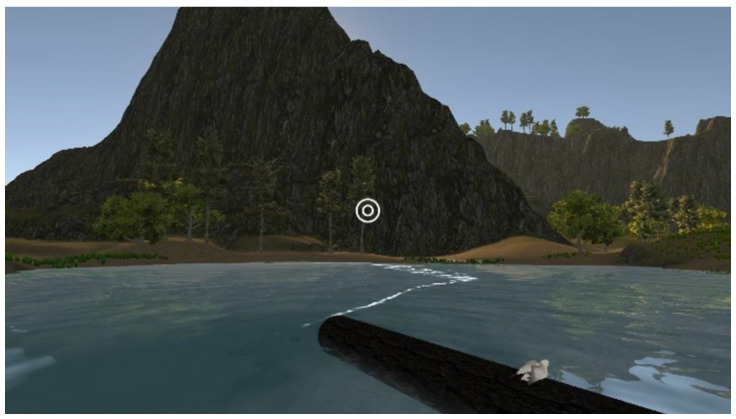
The first video clip presents 3D VR animation and human voices to tell a story about the content.

A storytelling video is played via 3D VR, which contains an ordering sequence of 15 video clips with 3D VR multimedia content. The Android app is developed to deal with the controls of playing the 3D VR video. As they start to use the app, players view the first video clip and at the same time listen to a human voice from the video. Figure 6 shows a screenshot of the first video clip simultaneously presenting 3D VR animation and human voices, telling a story about the content of the video clip. The design of the learning content follows multimedia and voice principles to offer rich information, i.e., 3D VR animation, videos, and human voices, rather than static graphics and text. Moreover, the 3D VR video consists of an ordering sequence of 15 video clips. This implements the segmentation principle.

The app provides interactive functions to build interactions between players and 3D VR multimedia content. The access procedures for these interactive actions are as follows.

**Figure 7:**
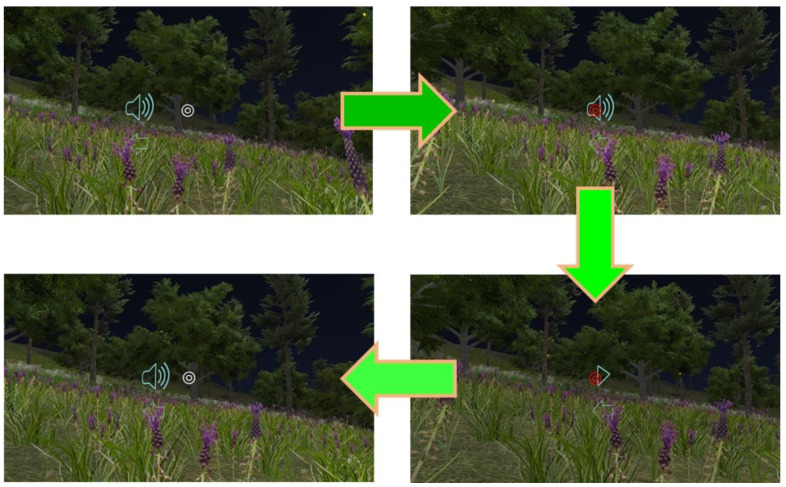
The procedure of replaying the current video clip.

**Figure 8:**
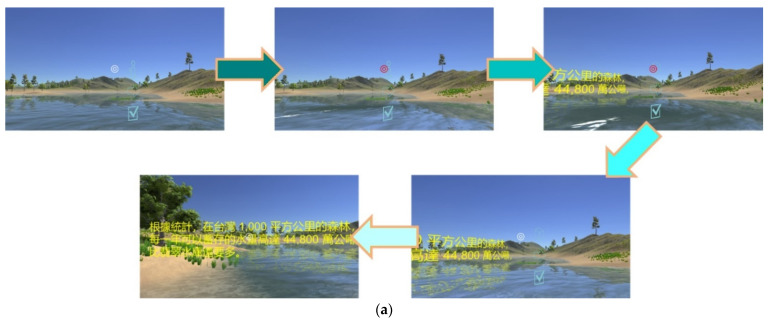

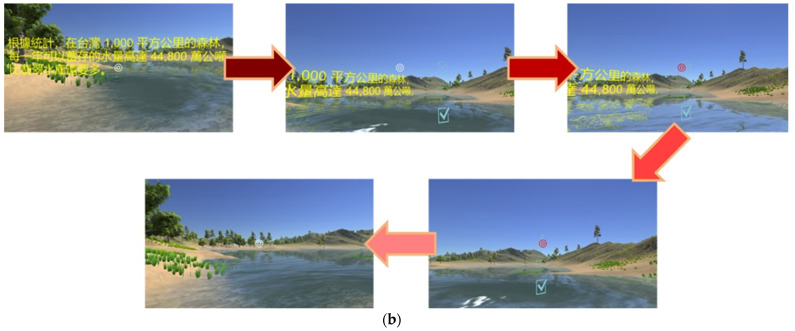
(**a**) The procedure of showing text annotations; (**b**) The procedure of hiding text annotations.

**Figure 9:**
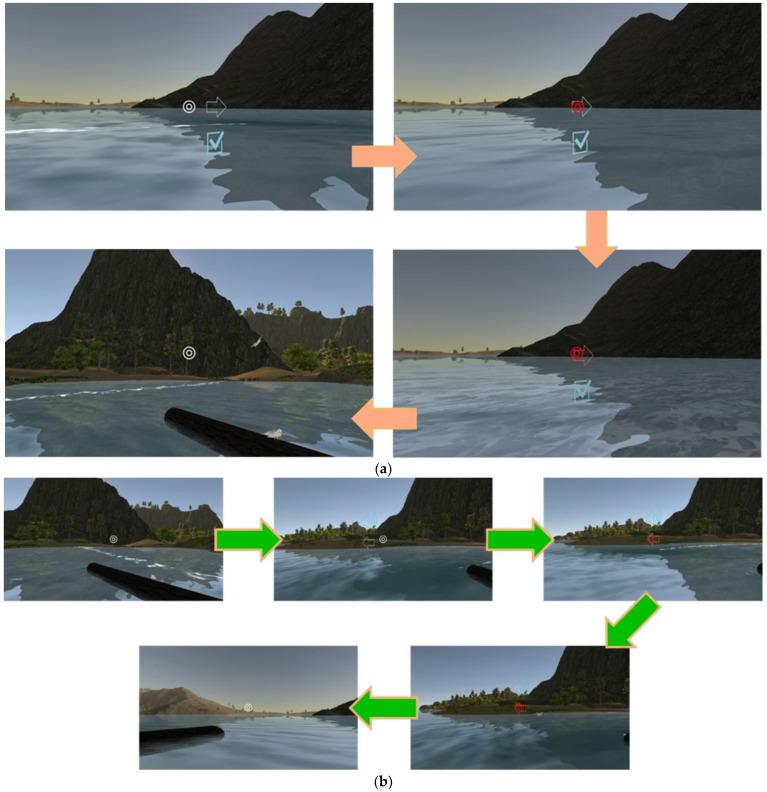
(**a**) Procedure for “going to next video clip.” (**b**) Procedure for “going to previous video clip.”

**Figure 10:**
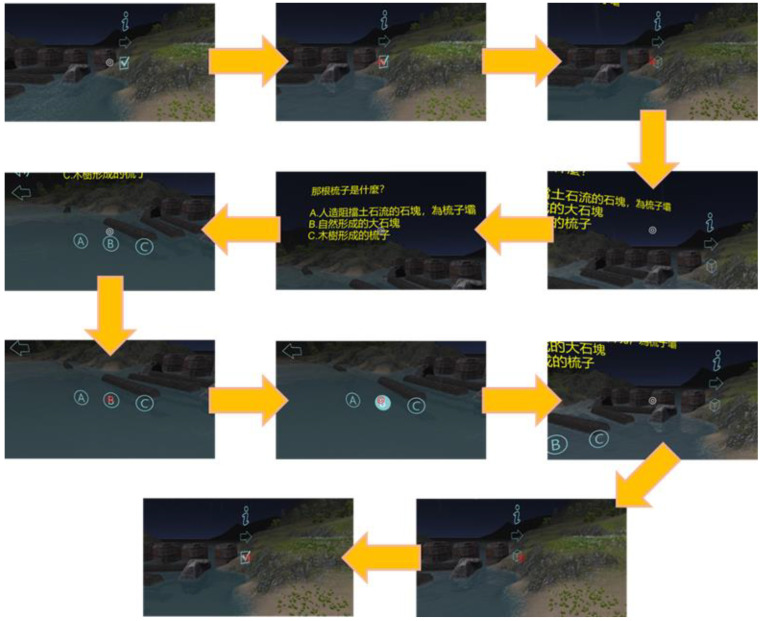
The procedure of displaying single choice questions first and then selecting an answer to questions.

1. Replaying the current video clip: Figure 7 illustrates the procedure for replaying the current video clip. Players move the white interaction sign to superimpose it on the sound icon. Once the interaction sign becomes red, it means that VRAM recognizes the trigger event and then carries out a replay of the current video clip. Consequently, the icon can be regarded as an anchor for the interaction action “replaying the current 3D VR video clip.” This implements the signaling principle by taking the sound icon as a cue to indicate a highlight in guiding players who can do the replaying operation. Here, the redundancy principle is implemented by simultaneously presenting 3D VR animation (graphic information) and spoken narration (auditory information). The modality principle states that humans learn better from animation with spoken narration than from animation with on-screen text. The video clip displays 3D VR animation without on-screen text. This also implements the coherence principle by displaying 3D VR animation as simpler visuals to enhance understanding. No irrelevant graphics, videos, animation, music, stories, or lengthy narration are provided in any video clip.
2. Showing/hiding text annotations: Players can make text annotations visible and invisible. Figure 8a shows the procedure of moving the interaction sign on the “i” icon to make the text annotation visible for reading. Figure 8b shows the procedure for hiding the visible text annotation. The visible text annotation becomes invisible automatically after an appropriate time (e.g., 10 s). This implements the redundancy and coherence principles by players who can hide visible text annotations or by the system making text annotations invisible automatically. Additionally, the spatial and temporal contiguity principles are implemented by offering simultaneous visual and text information. The design of each video clip realizes the aligning, related 3D VR animation and text annotations on the same screen. The authors of [20] showed that students’ learning efficacy could be enhanced if they received the use of annotations that can support the cognitive process for PowerPoint presentations in a classroom.
3. Going to the next video clip and going to the previous video clip: Figure 9a,b illustrates the procedure of performing these two interaction events, respectively. Two icons, “right arrow” and “left arrow,” indicate the events: “going to the next video clip” and “going to the previous video clip,” respectively. Players can switch between two consecutive video clips via triggering these two interaction events. This implements the segmentation principle by watching a multimedia lesson via user-paced segments rather than as a continuous unit. Players can employ these two icons to mimic a continue and replay button for controlling their favorite rate for watching each 3D VR video clip. Note that each video clip offers the replay function mentioned before. In Figure 5, regarding “going to next video clip” and “going to previous video clip,” these two events are recorded in xAPI format where the ordering number of each 3D VR video clip is saved in the page field.
4. Presenting a question and answering the question: Figure 10 illustrates the procedure of displaying single-choice questions first and then selecting an answer to questions. Players move the interaction sign by superimposing it on the icon, representing a tick within a square (or called a checkbox here), and then the cube icon appears to display a question, a single-choice item. Next, they move the interaction sign to superimpose it on one of the choice icons to complete answering the question. Finally, players move the interaction sign via superimposing it on the cube icon again to complete the action, hiding the visible question.

### 3.4. Learning Procedure Using VRAM

**Figure 11:**
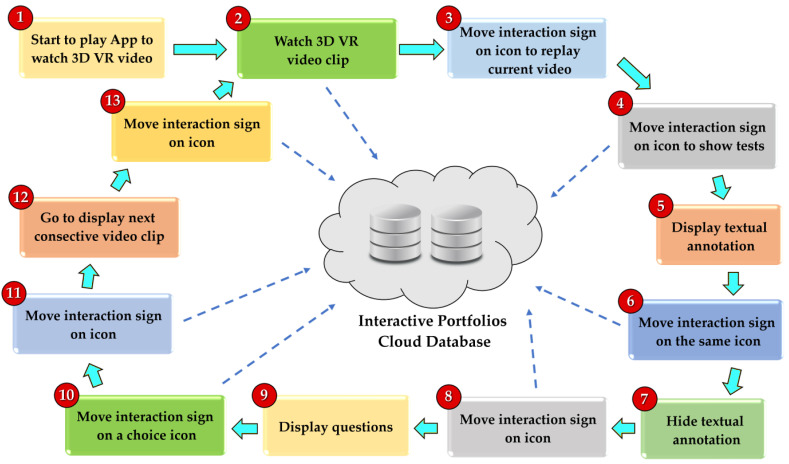
Learning procedure while watching the 3D VR video using a VRAM Android app.

Figure 11 displays an example of a learning procedure using VRAM while watching/playing the 3D VR video by an Android app. Dashed lines stand for sending interactive action records to the cloud to construct the interactive portfolios. Each step in Figure 11 is briefly given in Table 3. Additional hardware may be required, such as a Bluetooth button, for participants to use as “OK” or “Check” actions to trigger the events if shaking the head is unable to be performed due to disability.

### 3.5. Research Method, Collecting Samples, and Instrument

For the assessment of the perceived effectiveness of using VRAM in learning, an experiment was conducted on a university class here to carry out comparisons for participants’ flow experience, learning motivation, learning interaction, self-efficacy, and presence.

Participants at a university of science and technology in the middle of Taiwan enrolled in an IT class contributed to the experiment. Forty-four participants (38 males and 6 females) participated in the experiment and responded voluntarily to the invitation to fill out the questionnaire.

**Figure 12:**
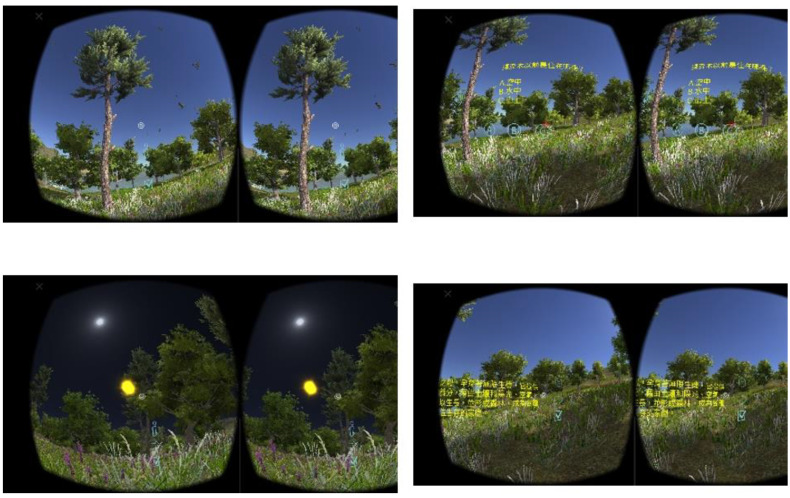
Views wearing VR helmets with a smartphone and an Android app to watch/play 3D VR content.

**Figure 13:**
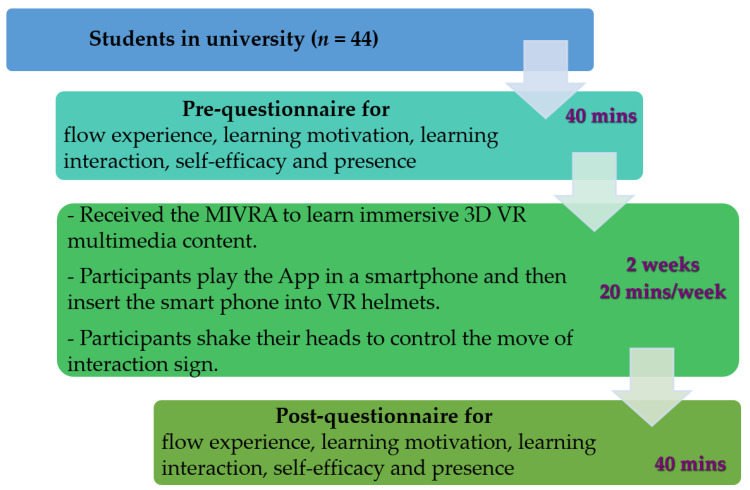
The 2-week experimental process.

A quasi-experimental study was conducted to examine the perceived effectiveness of using VRAM in learning 3D VR multimedia content. Figure 12 shows participants’ views while wearing the VR helmet with a smartphone playing the Android app to watch and/or play 3D VR multimedia content during the experimental process. Once participants trigger interactive actions, their interactive records are sent to the cloud immediately, as shown in Figure 5. The record is converted to xAPI format. Figure 13 displays the learning process of the 2-week experiment. Generally, the situation where participants wear the VR helmet cannot be too long. Therefore, the time period of the learning experiment took two weeks for watching 15 3D VR video clips. There is a need to carry out the a pretraining process of teaching participants to wear VR helmets, to play the Android app, and to trigger the interaction events that VRAM offers. After finishing the pretraining phase, they completed the prequestionnaires of flow experience, learning motivation, learning interaction, self-efficacy, and presence. After participants in the same group watched/played 15 3D VR video clips, they completed the postquestionnaires of flow experience, learning motivation, learning interaction, self-efficacy, and presence.

### 3.6. Measurement

The measurement tools used in the experiment comprised five measures for investigating the participants’ flow experience, learning motivation, learning interaction, self-efficacy, and presence. A 5-point Likert rating scheme was utilized in all questionnaires, where 1 and 5 expressed strongly disagree and strongly agree, respectively. Table 4 presents measurement sources and numbers of items for each measurement.

The flow-experience measurement was modified from that developed by referring to [30,31,32,33]. There are 14 items in this questionnaire that consist of five dimensions: 3 items for “Clear goal and feedback,” 3 items for “Concentrate on the task,” 3 items for “A sense of potential control,” 2 items for “Altered sense of time,” and 3 items for “The autotelic experience.” Cronbach’s alpha values for the questionnaire for before and after implementation were 0.803 and 0.922, respectively.

The learning motivation measurement was modified from that developed by referring to [34], and contains 18 items in this questionnaire. Cronbach’s alpha values for the questionnaire for before and after implementation were 0.916 and 0.944, respectively.

The learning interaction measurement was modified from that developed by referring to [35,36,37], and has 3 items in this questionnaire. Cronbach’s alpha values for the questionnaire for before and after implementation were 0.621 and 0.793, respectively.

The self-efficacy measurement was modified from that developed by referring to [35,38], and consists of 11 items in this questionnaire. Cronbach’s alpha values for the questionnaire for before and after implementation were 0.837 and 0.895, respectively.

The presence measurement was modified from that developed by referring to [35,39], and has 1 item in this questionnaire.

In Table 5, Cronbach’s alpha values for the questionnaire consisting of 5 measurements for before and after implementation were 0.953 and 0.974, respectively. From Table 5, the results reflect that the four measurements of the questionnaire mentioned above are good in reliability and internal consistency.

## 4. Experiment Results

Statistical paired *t*-tests were utilized in assessing the differences between pretest performance and posttest performance for participants. A paired sample was exploited to examine whether there were statistically significant differences for the five measurements of flow experience, learning motivation, learning interaction, self-efficacy, and presence for before and after implementation involving VRAM in learning 3D VR multimedia content. Here, the pair difference is defined by (after implementation–before implementation). Table 5 lists the statistical results. The mean values of these five measurements of learning effects for ratings on before and after implementation were 3.75 and 4.09, respectively. Standard error (SD) of ratings for before and after implementation were 0.488 and 0.640, respectively. Consequently, strong evidence demonstrates these five learning effects of participants had statistically significant increases (*t* = 2.914, *p* = 0.006).

For flow experience, the statistic reflected that participants’ flow experience had increased significantly (*t* = 2.546, *p* = 0.015). The learning motivation of participants was statistically significant (*t* = 2.808, *p* = 0.007). Concerning the learning interaction, the statistical results reflected that the learning interaction of participants had a significant increase, (*t* = 3.091, *p* = 0.003). In terms of self-efficacy, the statistical results revealed that participants’ self-efficacy had increased significantly (*t* = 2.712, *p* = 0.01). For presence, the statistical results indicated that participants’ presence had increased significantly (*t* = 3.483, *p* = 0.001).

In Table 6, regarding five dimensions of flow experience—clear goal and feedback, concentrate on the task, a sense of potential control, altered sense of time, and the autotelic experience—the statistical results reflected that four indicators of flow experience had increased significantly except for the indicator “altered sense of time.”

## 5. Discussion

In Table 6, the statistical results of the altered sense of time flow showed that participants’ altered sense of time was significantly improved. This indicator measures users’ perception of the transformation of time, i.e., players feel an altered sense of time. They may feel that time is either slowing down or speeding up [30,31,32]. The reason for the observation is that 15 3D VR video clips constitute the 3D VR video where each of them is presented by playing videos. Thereby, players cannot control the speed of each video clip. They merely skip the current 3D VR video clip via interaction events, going to next/previous clips. Consequently, players may feel that the watching time of each video clip cannot be altered, in contrast to the situation of playing games, which can be controlled by players while virtual objects in games are moving.

Regarding the indicators of learning interaction and a sense of potential control of flow, the results reflected that participants’ learning effectiveness on these two indicators showed highly significant improvements. The reason is that VRAM offers interactive actions, as shown in Figure 6, Figure 7, Figure 8, Figure 9 and Figure 10, which are useful interactions between participants and 3D VR multimedia content. Note that one of the main contributions of the paper is to provide these interactions in developing VRAM.

The authors of [39] stated that presence often indicates immersion into a virtual environment. In contrast, flow stands for an experience of immersion into a particular activity. The statistical results of the indications flow experience and presence revealed that participants’ flow experience and presence were significantly improved. Here, participants’ presence was superior to flow experience. The evidence of the situation may convey that VRAM provides a better immersive environment for watching/playing 3D VR multimedia content. In contrast, participants felt an insufficiently perceived flow of experience. This may point to the purpose of development of VRAM being exploited for watching 3D VR content, instead of playing games.

Due to producing 3D VR multimedia content based on principles of CTML, learning 3D VR multimedia content can enhance the participants’ cognition, i.e., learning in a 3D virtual environment for displaying 3D VR multimedia content emphasizes the important process of connecting the concepts and relationships with 3D VR animation, text annotations, narration with human voices, and responses to questions. Also, VRAM offers interaction actions triggered by participants in the virtual environment. The learning VR model VRAM shows the participants’ cognitive actions and possible choices while using the tool in the virtual environment. These two results show that using principles of CTML in developing VR content and models (environments) can promote human cognition are consistent with those concluded by [25].

Finally, by using the proposed VRAM framework, we believe that educational concepts can be delivered successfully in health-care systems, such as at the beginning of rehabilitation [40,41] or the stroke recovery process [42]. It can also be used for patients to acquire knowledge during rehabilitation.

However, several limitations still exist in this paper. First, only the Android version of the app was developed and applied in the learning procedure of the experimental process. Second, players’ head shaking may not be smooth in triggering interaction events. Third, watching 3D VR video clips for a long time may cause dizziness, nausea, motion sickness, or eye strain. In further research, an iOS version of the app will be developed. Additionally, players can employ the VR devices to control interaction events instead of shaking their heads. It is expected that other educational theories can be studied and applied in designing 3D multimedia content to enhance cognition. Finally, the high performance of 3D VR-displayed equipment can be quickly evolved to reduce the uncomfortable dizziness, nausea, motion sickness, and eye strain.

## 6. Conclusions

The paper has proposed VRAM, a mobile interaction application for playing 3D VR educational multimedia content, where the design of 3D VR multimedia content is based on CTML. The situation of watching/playing the 3D VR multimedia content is congruent with reading a picture book in Flash format for learning basic concepts of environmental conservation, i.e., a way of storytelling for reading 2D multimedia version of the picture book. Here, making 3D VR multimedia content followed CTML to enhance cognition. Learners wear the VR helmet to watch/play the 3D VR multimedia content (a sequential arrangement of 15 3D VR video clips). Shaking their heads can move the interaction sign by superimposing icons over 3D VR videos and then triggering interactive events. Meanwhile, these triggering actions are collected in the cloud immediately by the app and formatted by the experience API to form interactive portfolios. These portfolios can be analyzed in the future for understanding players’ behaviors when using VRAM.

A quasi experiment was conducted to realize an empirical study investigating the impact on the perceived learning effectiveness for participants’ flow experience, learning motivation, learning interaction, self-efficacy, and presence of employing VRAM in learning the 3D VR multimedia content. The statistical results of the questionnaire survey showed that with the help of exploiting VRAM for learning the 3D VR multimedia content, participants’ flow experience, learning motivation, learning interaction, self-efficacy, and presence were significantly promoted. Consequently, the VRAM developed in this paper is helpful in learning educational concepts for environmental conservation, such as restricted reservoir areas or the North Pole, to see climate change animation as well as dangerous places, such as landslides and rockslides, in the metaverse.

For future research, it may be interesting to add to the survey whether users have already used other technologies to aid teaching/learning, such as games, videos, and VR, as well as what they thought of their evolution in relation to our proposed VRAM.

## Figures and Tables

**Table 1:** A comparison for a color book as course material implemented by two methods.

Factors	High-Computation 3D VR Production Book	3D VR Color Book with App Version
Spread out quickly	No	Yes
Affordable hardware	No	Yes
3D VR environment	Yes	Yes
Interactions between users and 3D VR productions	Yes	Yes

**Table 2:** Overview of experimental studies on VR-based CTML for teaching and learning.

Study	VR Domain	Participants	VR Technology	Environments	Positive VR Effect
Daghestani et al., 2012 [25]	Simulations	104 students, 2nd grade students, ages 7–8	Java 3D (VRMM)	Virtual Desktop VR Interactive	Yes
Bhatti et al., 2017 [26]	Animation	None	3D Multimedia (ACTML)	3D Animation Interactive/PPT	Yes
Parong and Mayer, 2018 [27]	Immersion	55 college students, ages 18–30	VR, PPT Slides	HMD/Desktop PPT Slides	No
Wong and Notari, 2018 [19]	Immersion	None	AR/VR (eLEARN)	CMR/Mobile HoloLens	Yes
Meyer, Omdahl and Makransky, 2019 [28]	Immersion	118 students, ages 18–25, 60 students received immersive VR, 58 students received video	Immersive VR (CTML)	VR/Video	Yes

**Table 3:** Steps for actions in Figure 11.

Steps	Description
**1**	Start to watch the 3D VR video using an Android app in a smartphone.
**2**	Watch the current 3D VR video clip, as shown in Figure 6.
**3**	Movie interaction sign on the sound icon to trigger the event “replay current video clip,” as shown in Figure 7.
**4**	Movie interaction sign on the “i” icon to trigger the event “display text annotation,” as illustrated in Figure 8a.
**5**	Display corresponding text annotation for the “i” icon, as exhibited in Figure 8a.
**6**	Move the interaction sign on the “i” icon again to trigger the event “hide text annotation,” as displayed in Figure 8b.
**7**	Hide corresponding text annotation for the “i” icon, as shown in Figure 8b.
**8**	Move the interaction sign on the checkbox icon, ☑, to trigger the event “display a question,” as shown in Figure 10.
**9**	Display a question, as illustrated in Figure 10.
**10**	Move the interaction sign on the icon, one of the choices, to select an answer to the question, as shown in Figure 10.
**11**	Move the interaction sign on the right-arrow icon, as shown in Figure 9.
**12**	Play next video clip, as exhibited in Figure 9.
**13**	Move interaction sign on the left-arrow icon, as illustrated in Figure 9.
**2**	Back to watch current video clip, as shown in Figure 9.

**Table 4:** Measurement sources and numbers of items.

Measurements (Dimensions)	Sources
Flow experience (14) • Clear goal and feedback (3) • Concentrate on the task (3) • A sense of potential control (3) • Altered sense of time (2) • The autotelic experience (3)	Csikszentmihalyi,1975 [30]; Csikszentmihalyi, 1990 [31]; Csikszentmihalyi, 1993 [32]; Guo, 2004 [33]
Learning motivation (18)	Wei et al., 2015 [34]
Learning interaction (3)	Merchant, et al., 2012 [35]; Moore, 1989 [36]; Siau, Sheng and Nah, 2006 [37]
Self-efficacy (11)	Merchant, et al., 2012 [35]; Witt-Rose, 2004 [38]
Presence (1)	Merchant, et al., 2012 [35]; Lee, Wong and Fung, 2010 [39]

**Table 5:** Internal consistency analysis with quantitative data.

Measurements	Cronbach’s Alpha	
	Before Implementation	After Implementation
Flow experience	0.803	0.922
Learning motivation	0.916	0.944
Learning interaction	0.621	0.793
Self-efficacy	0.837	0.895
All	0.953	0.974

**Table 6:** Hypothesis testing of paired *t*-test results related to flow experience, learning motivation, learning interaction, self-efficacy, and presence.

Research Questions	*t*-Value	d.f.	*p*-Value
Flow experience	2.546	43	0.015
Flow experience—Clear goal and feedback	2.864	43	0.006
Flow experience—A sense of potential control	2.806	43	0.007
Flow experience—Altered sense of time	1.091	43	0.281
Flow experience—Autotelic experience	2.799	43	0.008
Learning motivation	2.808	43	0.007
Learning interaction	3.091	43	0.003
Self-efficacy	2.712	43	0.010
Presence	3.483	43	0.001
All	2.914	43	0.006

## Data Availability

Not applicable.
